# Fetal exposure to paracetamol is associated with altered markers of ovarian development and reduced uterine volume in girls: the COPANA study

**DOI:** 10.1093/hropen/hoag073

**Published:** 2026-09-08

**Authors:** Margit B Fischer, Gylli Mola, Karin Sundberg, Lone Scheel, Katrine B Wraae, Ane L Rom, Hanne Frederiksen, Richard A Anderson, Maria Assens, Anna-Maria Andersson, Lærke Priskorn, Jørgen H Petersen, Hanne K Hegaard, Katharina M Main, David M Kristensen, Anders Juul, Casper P Hagen

**Affiliations:** Department of Growth and Reproduction, Rigshospitalet, Copenhagen, Denmark; International Center for Research and Research Training in Endocrine Disruption of Male Reproduction and Child Health (EDMaRC), Rigshospitalet, Copenhagen, Denmark; Department of Growth and Reproduction, Rigshospitalet, Copenhagen, Denmark; International Center for Research and Research Training in Endocrine Disruption of Male Reproduction and Child Health (EDMaRC), Rigshospitalet, Copenhagen, Denmark; Department of Obstetrics, Center of Fetal Medicine and Pregnancy, Rigshospitalet, Copenhagen, Denmark; Department of Obstetrics, Center of Fetal Medicine and Pregnancy, Rigshospitalet, Copenhagen, Denmark; Department of Obstetrics, Center of Fetal Medicine and Pregnancy, Rigshospitalet, Copenhagen, Denmark; Department of Gynaecology, Fertility and Obstetrics, Rigshospitalet, Copenhagen, Denmark; Research Unit of Gynaecology and Obstetrics, Department of Clinical Research, University of Southern Denmark, Odense, Denmark; Department of Growth and Reproduction, Rigshospitalet, Copenhagen, Denmark; International Center for Research and Research Training in Endocrine Disruption of Male Reproduction and Child Health (EDMaRC), Rigshospitalet, Copenhagen, Denmark; Centre for Reproductive Health, University of Edinburgh, Edinburgh, UK; Department of Growth and Reproduction, Rigshospitalet, Copenhagen, Denmark; International Center for Research and Research Training in Endocrine Disruption of Male Reproduction and Child Health (EDMaRC), Rigshospitalet, Copenhagen, Denmark; Department of Growth and Reproduction, Rigshospitalet, Copenhagen, Denmark; International Center for Research and Research Training in Endocrine Disruption of Male Reproduction and Child Health (EDMaRC), Rigshospitalet, Copenhagen, Denmark; Department of Growth and Reproduction, Rigshospitalet, Copenhagen, Denmark; International Center for Research and Research Training in Endocrine Disruption of Male Reproduction and Child Health (EDMaRC), Rigshospitalet, Copenhagen, Denmark; Department of Growth and Reproduction, Rigshospitalet, Copenhagen, Denmark; Department of Biostatistics, University of Copenhagen, Copenhagen, Denmark; Department of Gynaecology, Fertility and Obstetrics, Rigshospitalet, Copenhagen, Denmark; Department of Clinical Medicine, University of Copenhagen, Copenhagen, Denmark; Department of Growth and Reproduction, Rigshospitalet, Copenhagen, Denmark; International Center for Research and Research Training in Endocrine Disruption of Male Reproduction and Child Health (EDMaRC), Rigshospitalet, Copenhagen, Denmark; Department of Clinical Medicine, University of Copenhagen, Copenhagen, Denmark; Department of Growth and Reproduction, Rigshospitalet, Copenhagen, Denmark; Department of Science and Environment, Roskilde University, Roskilde, Denmark; Department of Growth and Reproduction, Rigshospitalet, Copenhagen, Denmark; International Center for Research and Research Training in Endocrine Disruption of Male Reproduction and Child Health (EDMaRC), Rigshospitalet, Copenhagen, Denmark; Department of Biostatistics, University of Copenhagen, Copenhagen, Denmark; Department of Growth and Reproduction, Rigshospitalet, Copenhagen, Denmark; International Center for Research and Research Training in Endocrine Disruption of Male Reproduction and Child Health (EDMaRC), Rigshospitalet, Copenhagen, Denmark; Department of Biostatistics, University of Copenhagen, Copenhagen, Denmark

**Keywords:** paracetamol, acetaminophen, prenatal exposure, fetal exposure, female reproductive health, minipuberty, ovarian development

## Abstract

**STUDY QUESTION:**

Is fetal exposure to paracetamol associated with markers of ovarian function in infancy?

**SUMMARY ANSWER:**

Mild to moderate doses of prenatal paracetamol exposure, assessed by detailed maternal reports and urinary measurements, are associated with ovarian morphology and activity as well as reduced size of estrogen-responsive tissues in infant girls.

**WHAT IS KNOWN ALREADY:**

Maternal use of paracetamol is widespread. Across multiple independent animal studies, fetal exposure consistently impairs the formation of primordial ovarian follicles, causing subfertility and premature estropause in female offspring.

**STUDY DESIGN, SIZE, DURATION:**

The Copenhagen Analgesic study (COPANA) is a single-center, prospective, observational cohort study conducted at a university hospital (March 2020 to November 2022). From 3425 eligible participants, 685 healthy singleton pregnant women of Caucasian origin, were enrolled in the first trimester of pregnancy, and 302 infant daughters were examined at follow-up. Exclusion criteria included maternal diabetes or thyroid disease, pre- or post-term delivery, or severe infant illness.

**PARTICIPANTS/MATERIALS, SETTING, METHODS:**

Pregnant women reported paracetamol use biweekly and provided first-trimester urinary samples which were analyzed for paracetamol levels (LC-MS/MS) and adjusted for urinary osmolarity (n = 299). Girls were classified by timing of initial exposure: early fetal life (<17 weeks, n = 92), mid–late fetal life (≥17 weeks, n = 67), or unexposed controls (n = 143). Of the 92 girls initially exposed in early fetal life, a subgroup was exposed exclusively in early fetal life (n = 22).

In an independent confirmatory cohort, 1210 girls were followed from infancy to adolescence. Their exposure was maternal self-reported (any) use of paracetamol during pregnancy and reported early in the third trimester (yes/no).

**MAIN RESULTS AND ROLE OF CHANCE:**

Early fetal exposure was associated with reduced ovarian volume (−0.11 cm^3^, 95% CI −0.19 to −0.03) and reduced uterine volume (−0.18 cm^3^, −0.35 to −0.01), whereas mid–late fetal exposure was associated with fewer ovarian follicles (−1.05 follicles, −1.71 to −0.39) compared to unexposed girls. AMH levels were lower in girls exposed exclusively in early fetal life (−0.45 SDS, −0.87 to −0.03) compared to unexposed girls. Maternal urinary paracetamol concentrations were inversely associated with ovarian and uterine volume as well as breast tissue diameter. In the independent confirmatory cohort, fetal paracetamol exposure was associated with reduced uterine volume at puberty (−4.11 cm^3^, −7.29 to −0.92) and smaller ovarian volume during adolescence (−2.76 cm^3^, −4.82 to −0.70).

**LIMITATIONS, REASON FOR CAUTION:**

The observational design of the study allows evaluation of exposure outcome associations, while the causality interpretation is supported by consistency with experimental models demonstrating comparable effects. Residual confounding by indication for paracetamol use cannot be completely excluded, although the results were robust after accounting for fever and other maternal factors. The analytic design of the current study limited our ability to evaluate whether frequency or patterns of paracetamol use influenced the observed associations.

**WIDER IMPLICATIONS OF THE FINDINGS:**

In this prospective cohort study of infant girls, prenatal exposure to paracetamol was associated with differences in several markers of ovarian development, including ovarian volume, follicle number, and circulating AMH, as well as uterine volume and breast tissue diameter. The associations with reduced ovarian and uterine size were also observed in a confirmatory cohort followed to adolescence. Whether these early-life differences have long-term clinical relevance, including effects on reproductive lifespan, remains uncertain and requires analyses in studies with extended follow-up.

**FUNDING:**

This research was supported by Rigshospitalets Research Council under grant (E-22717-21), Læge Sofus Carl Emil Friis og hustru Doris Friis‘Legat (F-23936-01), Aase og Ejnar Danielsens Foundation (20-10-0367), Helsefonden (20-B-0388), Axel Muusfeldt Foundation (2020-0385), and The Danish Centre for Endocrine Disrupting Substances (CeHoS) (2022-23219). The funders had no role in considering the study design or in the collection, analysis, interpretation of data, writing of the report, or decision to submit the article for publication. The publication was completed as part of the MERLON project under grant agreement No. 101137411. The MERLON project is funded by the European Union. Views and opinions expressed are, however, those of the authors only and do not necessarily reflect those of the European Union or the European Health and Digital Executive Agency (HADEA). Neither the European Union nor HADEA can be held responsible for them.

**DISCLOSURES:**

All authors declare no support from any organization for the submitted work, no financial relationships with any organizations that might have an interest in the submitted work in the previous 3 years, and no other relationships or activities that could appear to have influenced the submitted work. One author (MA) is a government official.

**TRIAL REGISTRATION NUMBER:**

ClinicalTrials.gov ID: NCT0436922.

WHAT DOES THIS MEAN FOR PATIENTS? Paracetamol is bought over the counter and is used to treat mild pain and fever. It is commonly used during pregnancy and is generally considered safe. However, there is limited knowledge about whether exposure during fetal life may affect the development of the female reproductive system.

In this study, 302 healthy girls from a Danish pregnancy cohort were examined during infancy. Paracetamol exposure during pregnancy was assessed using both pregnant women’s reports of paracetamol use collected bi-weekly and measurements of paracetamol in their urine samples.

We found that girls who had been exposed to paracetamol during fetal life had smaller ovaries, fewer ovarian follicles (the structures that contain immature eggs), differences in reproductive hormone levels, and reduced uterine size during infancy. Similar findings were observed in an independent group of participants followed to adolescence.

This is the first human study specifically designed to investigate whether prenatal paracetamol exposure is associated with markers of ovarian development in daughters. While this study cannot prove that paracetamol directly caused these differences, the findings are consistent with results from animal studies. Further research is needed to determine whether these early-life differences have consequences for reproductive health later in life and to inform recommendations on paracetamol use during pregnancy.

## Introduction

The foundation of adult reproductive function is established *in utero* through the development and differentiation of the fetal gonads. Females are born with a defined number of primordial follicles that deplete throughout their reproductive lifespan inevitably leading to menopause (Baker, 1963; Wallace and Kelsey, 2010). Fetal establishment of the primordial follicle pool is therefore crucial for female reproductive health.

In early fetal life, oogenesis involves rapid germ cell proliferation and the formation of primordial follicles in gestational weeks (GWs) 10–17 (Baker, 1963; Forabosco and Sforza, 2007). The transition from mitosis to meiosis and subsequent arrest in prophase I is critical for oocyte survival and for establishing the follicle pool (Hartshorne *et al.*, 2009).

From mid-to-late fetal life, mitosis declines as more germ cells enter meiosis and folliculogenesis begins (Kurilo, 1981; Forabosco and Sforza, 2007). These sequential developmental phases represent highly sensitive windows in which adverse exposure may have long-term consequences for female reproductive health.

Minipuberty is a transient activation of the hypothalamic–pituitary–gonadal (HPG) axis during infancy resulting in increased ovarian activity including ovarian follicle growth and feedback mechanisms resembling those of adult women (Ljubicic *et al.*, 2022; Fischer *et al.*, 2025). This provides a unique opportunity to assess ovarian activity in early infancy.

Paracetamol (*N*-acetyl-*p*-aminophenol, otherwise known as acetaminophen) is sold over the counter, reducing its perception as a pharmacologic agent with potential side effects (Bauer *et al.*, 2021). Due to its analgesic and antipyretic properties, it is used by more than 50% of women during pregnancy, and it passes through the placenta freely (Nitsche *et al.*, 2017).

In rodent studies, fetal paracetamol exposure reduces ovarian follicle reserve by up to 40% in female offspring, leading to impaired fertility and shortened reproductive lifespans (Dean *et al.*, 2016; Holm *et al.*, 2016; Johansson *et al.*, 2016; Hurtado-Gonzalez *et al.*, 2018; Rossitto *et al.*, 2019; Aleixo *et al.*, 2020; Wu *et al.*, 2024).

Comparable findings have been observed with acetylsalicylic acid and other non-steroidal anti-inflammatory drugs, likely due to their irreversible inhibition of prostaglandin synthases (Kristensen *et al.*, 2012; Mazaud-Guittot *et al.*, 2013). Acetylsalicylic acid is commonly prescribed during pregnancy to women at risk of complications (Roberge *et al.*, 2018).

Beyond pharmaceutical use, paracetamol exposure may also occur environmentally through aniline, an industrial precursor found in textiles and pesticides, which is metabolized to paracetamol in the liver (Kristensen *et al.*, 2016; Pot *et al.*, 2022). Previous biomonitoring studies have reported measurable urinary paracetamol (U-paracetamol) concentrations in the general population even in the absence of obvious paracetamol exposure (Modick *et al.*, 2014). Urinary paracetamol levels provide an objective biomarker of recent exposure and complement self-reported medication.

While experimental and animal data consistently demonstrate the harmful effects of fetal exposure to paracetamol on gonadal development, evidence in humans remains limited. The Copenhagen Analgesic Study, COPANA, is the first human study primarily designed to evaluate whether fetal exposure to paracetamol is associated with impaired gonadal function. We assessed ovarian and uterine morphology as well as circulating levels of reproductive hormones in 302 infant girls during minipuberty. Findings were further evaluated in an independent confirmatory cohort, The Copenhagen Mother-Child Cohort, followed from fetal life through adolescence (Chellakooty *et al.*, 2003; Hagen *et al.*, 2015; Assens *et al.*, 2020).

## Materials and methods

### Population

The COPANA study is a single-center, prospective, observational cohort study conducted at the Copenhagen University Hospital—Rigshospitalet, Denmark (March 2020 to November 2022) (Fischer *et al.*, 2024b).

The COPANA Study (ClinicalTrials.gov ID: NCT04369222) adhered to STROBE Guidelines (Vandenbroucke *et al.*, 2007) and was approved by the ethical committee of the Capital Region of Denmark (H-19044036) and the Danish Data Protection Agency (P-2019-330). Participants gave written informed consent. For infant participants, consent was obtained from all adults with legal guardianship.

A total of 3425 healthy pregnant women (Caucasian, BMI 18–35 kg/m^2^, with no pre-existing diabetes or thyroid disease) were invited to participate after completing a routine web-based questionnaire (Q1) providing the obstetrics department with patient-reported health information. From these, 685 women were enrolled. To obtain a homogeneous study population, and to avoid conditions possibly affecting fetal development and hormone levels, women were excluded due to multiple pregnancy, gestational diabetes, thyroid disease, or pre- or post-term delivery (< 37 GW or > 42 GW), or serious illness in the infant such as genetic conditions and birth defects. Women reporting substance use (other than nicotine or alcohol) were also excluded. Further, at inclusion, all women were asked to complete a questionnaire on general and reproductive health (Q2).

Participating families visited three times during the study: in the first trimester, at a third trimester fetal ultrasound scan, and for an infant examination ∼3 months postpartum during minipuberty (n = 302 girls). For the study flowchart and timeline, see Supplementary Fig. S1.

### Exposure to paracetamol

The Q1 questionnaire from which eligible women were invited was completed in mean (±SD) GW 9.8 (2.4) and entailed information about current use of mild analgesics (‘Do you currently take any mild analgesic?’ (Consumption: yes/no, rarely/1–2 times weekly/daily, drug name). From the time of inclusion, GW 14.1 (1.8), and throughout the study period, the pregnant women completed detailed electronic questionnaires (Q3) every 2 weeks concerning medicine consumption with a focus on mild analgesics (consumption: yes/no, drug name, dose per use (mg), indication). The first questionnaire on medicine consumption at inclusion covered the period from the invitation to inclusion. Detailed information on medicine intake is therefore available from completion of Q1 at GW 9.8 (2.4). The specific generic and brand names of all available mild analgesic products were listed in the questionnaire. After birth, the questionnaire included questions about medication given to the infant.

Two fetal exposure periods were predefined reflecting different essential aspects of follicle formation and thus potentially different vulnerability regarding exposure.

Exposure in early fetal life included maternal use of paracetamol from invitation to GA 17 weeks. This period is characterized by intense mitotic proliferation of the oogonia, followed by initiation of the first meiotic division.

Exposure in mid–late fetal life included maternal use of paracetamol in GA ≥ 17 weeks, a period where mitotic activity subsides, paralleled by an increasing rate of meiotic entry. Moreover, folliculogenesis is ongoing.

We grouped the girls according to initiation of exposure within the predefined exposure periods.

Girls who were not exposed to paracetamol during fetal life were classified as ‘unexposed controls’. A subgroup of girls was exposed to paracetamol exclusively in early fetal life. Girls initially exposed in mid–late fetal life were, by definition, exclusively exposed in this period. In addition to self-reported paracetamol use, the pregnant women provided a urine sample at the inclusion visit, which was analyzed for urinary levels of paracetamol.

### Third trimester ultrasound scan

At GW 29–34, all included pregnant women were offered a study-specific ultrasound scan (Voluson E10, GE HealthCare). Fetal anogenital distance (AGD) was measured in the axial plane from the center of the anus to the posterior converge of the fourchette by a board-certified technician (LS) with at least three repeated measurements wherefrom the average was calculated.

### Infant examination

All infants were examined during minipuberty (age mean (±SD) 106 days (13)) with weight and length measurements. AGDaf (ano-fourchettal) and AGDac (ano-clitoral) were measured using the TIDES method (Sathyanarayana *et al.*, 2015). Breast tissue was assessed by palpation, and the diameter was measured with a ruler.

A transabdominal ultrasound scan (TAUS) was performed by a single experienced clinician (MBF), blinded to exposure status (Voluson E10, GE HealthCare). Girls were scanned with a full bladder in a supine position with a slight incline. One or both ovaries were identified in 203 girls (bilaterally in 136 girls and unilaterally in 67 girls (left: n = 22, right: n = 45)). The length (L) and width (W) were measured, and ovarian volume was calculated (L × W × W × π/6) separately for the left and right side, whereafter an average of the two measures was used when both ovaries were identified. Antral follicles larger than 2 mm were counted. The sum of follicles from both ovaries was used as the outcome.

Left- and right-side ovarian volume (r = 0.48, *P* < 0.001, n = 136, Spearman′s rho) and follicle count (r = 0.38, *P* < 0.001, n = 136) correlated positively. Therefore, in cases where only one ovary was visualized, we used that ovarian volume or follicle count (doubled) as representative data for the contralateral ovary.

The uterus was scanned in the sagittal view, where the uterine length (SAG, including the cervix) was measured and at the widest point, the anteroposterior width (AP) and the thickness of the endometrium. In the transverse view, the widest transverse diameter (TRV) of the uterine fundus was measured. Uterine volume was calculated (SAG × AP × TRV × π/6).

### Serum and urine samples

Concentrations of reproductive hormones were measured from blood samples drawn from an antecubital vein (n = 269/302, 89%). Samples were clotted and centrifuged, and serum was stored at −20°C until analysis. The peptide hormones, anti-Müllerian hormone (AMH), follicle-stimulating hormone (FSH), luteinizing hormone (LH), and inhibin B were analyzed using immunoassays.

The concentration of estradiol was analyzed in-house by isotope-diluted online-TurboFlow-liquid chromatography mass spectrometry (LC-MS/MS) (Frederiksen *et al.*, 2020a). For details on assays, detection limits (LOD) and prioritization of analyses, see Supplementary Table S1.

Maternal urine samples (n = 299/302 (99%)) were analyzed for the total (free and conjugated) concentration of paracetamol in-house by isotope-diluted LC-MS/MS with prior enzymatic deconjugation and adjusted for urinary dilution by osmolality adjustments, according to previous description (Frederiksen *et al.*, 2020b). LOD for paracetamol was 0.48 µg/l.

### Outcomes

Primary outcomes were predefined as markers of ovarian function: ovarian volume, number of follicles, and circulating levels of AMH (Fischer *et al.*, 2024b), as well as uterine size and glandular breast tissue diameter (Fischer *et al.*, 2024a).

Secondary outcomes were predefined as key parameters relating to the primary outcomes, e.g. AGD and endometrial thickness, as well as circulating levels of FSH, LH, and inhibin B.

### Statistics

Continuous data were summarized using mean (±SD) or median (IQR) as appropriate and compared between exposure groups using Student’s *t*-test or Mann–Whitney *U*-test, while categorical data were summarized as frequency (%) and compared using the chi-square test or Fisher’s exact test when expected cell counts were ≤ 5. To adjust infant hormone concentrations for sex and age at measurement, we calculated sex- and age-specific standard deviation scores (SDS). We used reference curves from a previous extensive longitudinal study (Busch *et al.*, 2021) which were created using the generalized additive model for location, scale, and shape (GAMLSS) including the GAMLSS R package (Rigby and Stasinopoulos, 2014). Limited serum volumes occasionally prevented full hormone analysis. Values below the LOD were assigned as LOD/2. LH was above LOD in just 47% of cases and therefore dichotomized to above LOD (yes/no).

We performed linear regression analyses to estimate regression coefficients (β) and 95% CIs for the association between pre-specified exposure windows (early/mid–late) and predefined outcomes (dependent variables) compared to unexposed controls. To assess a possible dose–response trend, accumulated maternal self-reported doses of paracetamol use during pregnancy, as well as urinary paracetamol concentrations, were evaluated by generating binary exposure variables at percentile thresholds (at 1% increments) ranging from the 50th to the 95th percentile. For each cutoff, participants above the threshold were compared with those below using linear regression models. Plots of residuals and QQ plots were used to assess model assumptions. Because residual diagnostics indicated that one variable (ovarian volume) did not fully meet the assumptions of normality and homoscedasticity, all regression analyses were conducted using robust variance estimators, which leave point estimates unchanged but provide heteroskedasticity-consistent standard errors, yielding more reliable CIs and significance tests. This approach also allowed the outcome variables to be analyzed on their original scales without transformation, facilitating the interpretation of regression coefficients in their natural units.

Regression analyses were performed unadjusted as well as adjusted. Covariates were selected *a priori* based on biological plausibility and prior experimental evidence, i.e. maternal use of acetylsalicylic acid (Mazaud-Guittot *et al.*, 2013) and other non-steroidal anti-inflammatory drugs (Leverrier-Penna *et al.*, 2018) and infant treatment with paracetamol (Ndeke *et al.*, 2022), maternal self-reported diagnosis of polycystic ovarian syndrome (Barrett *et al.*, 2018), and maternal consumption of alcohol in the first trimester (Weinberg *et al*., 2008), or observed differences between exposure groups, i.e. maternal pre-pregnancy BMI, maternal use of nicotine products in the first trimester, and conception by IVF. Moreover, fetal AGD was adjusted for gestational age at the time of ultrasound scan, and measurements of infant AGD, uterine sizes, and breast tissue diameter were adjusted for infant age at the time of examination.

Using multiple linear regression, we estimated the independent effect of exposure in each fetal period on the outcome variable while accounting for potential overlap between exposure periods. For each exposure period, a ‘dummy variable’ was created, representing any paracetamol use during that specific period (yes/no), regardless of exposure in other fetal periods. The regression model included both dummy variables (early/mid–late fetal exposure) as independent variables simultaneously.

To assess the robustness of the observed associations, we conducted a series of sensitivity analyses. Specifically, we repeated the univariate regression models while sequentially excluding infants based on the presence of each binary (yes/no) covariate at a time.

To assess the degree of confounding by indication, we conducted an additional univariate analysis excluding infants from mothers who reported fever as the indication for paracetamol use during pregnancy.

A two-sided *P*-value was considered statistically significant at *P* ≤ 0.05. Trends were noted for *P*-values between 0.05 and 0.1. All statistical analyses were performed using IBM SPSS Statistics 22.0 (IBM Corp., 2013. IBM SPSS Statistics for Windows, Version 22.0. Armonk, NY, USA: IBM Corp., n.d.), and RStudio (RStudio Team (version 2024.04.2). RStudio: Integrated Development for R. RStudio, PBC, Boston, MA, USA).

### Independent confirmatory cohort

The Copenhagen Mother-Child Cohort is a population-based, longitudinal, prospective pregnancy, and birth cohort initiated in 1996 with inclusion of pregnant women from three university hospitals in the greater Copenhagen area. Only pregnant women with Danish-born and -raised parents and grandparents were included. In total, 1210 girls born between 1997 and 2002 were included at birth (Chellakooty *et al.*, 2003).

#### Exposure

While this cohort was not primarily designed to evaluate associations between gonadal function and fetal exposure to mild analgesics, information on maternal use of medication during pregnancy was collected by questionnaire at the beginning of the third trimester. Mothers were asked if they had used any form of medication during pregnancy (yes/no/do not know), and paracetamol was specifically listed as an example. We grouped girls according to exposure to paracetamol and/or NSAIDs at any point during pregnancy (n = 179) versus unexposed controls including those answering, ‘do not know’ (n = 1012).

#### Examinations

Girls were examined at infancy (n = 1083, age mean (±SD) 101 (24) days), at puberty (n = 563, age 10.7 (1.5) years), and at adolescence (n = 317, age 16.05 (0.89) years). At all examinations, girls underwent blood sampling and at puberty and adolescence, pubertal development was assessed by Tanner staging (Marshall and Tanner, 1969).

At puberty, a subset of girls (n = 121) had a magnetic resonance imaging (MRI) of the uterus and ovaries. Magnetic resonance imaging was performed with a 3-Tesla MRI (Magnetom Verioto Siemens AG) as previously described (Hagen *et al.*, 2015). At adolescence, a TAUS of the uterus and ovaries (n = 317) was performed (Voluson E8 Ultrasound System (GE Healthcare Medical Systems, Zipf, Austria)) (Assens *et al.*, 2020). Adolescent girls were examined during cycle day 2–5 when possible. For both MRI and TAUS, the uterus was measured in three planesto sagittal (SAG), width (AP), and transverse (TRV) and the volume was calculated assuming an ellipsoid shape (SAG × AP × TRV × π/6). Likewise, ovarian volume was calculated using the length, width, and transverse length. The number of follicles was counted in each ovary, and the total follicle count was calculated as the sum of follicles from both ovaries.

#### Serum samples

Serum FSH and LH were measured in infancy by Delfia (Wallac, Inc, Turku, Finland, LODs = 0.06 and 0.05 IU/liter, respectively) (Chellakooty *et al.*, 2003), and in puberty and adolescence by Delfia (PerkinElmer, Boston, MA, USA, LOD = 0.05 IU/l) (Hagen *et al.*, 2015; Assens *et al.*, 2020). Serum inhibin B was measured in infancy by an enzyme-linked immunosorbent assay (ELISA, Oxford Bio-Innovation, UK, LOD = 20 pg/ml) (Chellakooty *et al.*, 2003), and in puberty and adolescence by the Beckman Coulter GenII assay (Beckman Coulter, Inc. Brea, CA, USA, LOD = 3.0 pg/ml).

In infancy and puberty, AMH was measured using the Beckman Coulter enzyme immunometric assay generation I (IOT, Immunotech, Beckman Coulter, Marseille, France) with a detection limit of 2.0 pmol/l.

During the follow-up of adolescent girls, the Access immunoassay substituted the Immunometric assay generation I when this was no longer commercially available. The Access immunoassay (Beckman Coulter, Brea, CA, USA) has a detection limit of 0.14 pmol/l. Comparative studies between the two assays revealed negligible differences (1.3%), and no adjustments of results have therefore been performed.

#### Statistics

We performed linear regression analyses to estimate regression coefficients (β) and 95% CIs for the association between serum sample analyses as well as uterine and ovarian morphology (puberty and adolescence) (dependent variables) and exposure compared to unexposed controls (yes/no) (independent variable). Pubertal outcomes were adjusted for tanner stage at the time of the examination, and for adolescent outcomes, all girls using hormonal contraceptives were excluded. To ensure comparability with the COPANA cohort, we additionally adjusted for GW at birth and maternal thyroid disease or diabetes at all examination points (infancy/puberty/adolescence).

The Copenhagen Mother-Child cohort was conducted according to the Helsinki II declaration and approved by the local Ethical Committee (reference no. (KF) 01-030/97) and the Danish Registry Agency (journal no. 1997-1200-074).

## Results

### COPANA cohort

In total, use of paracetamol during pregnancy was reported by 159 women (53%) whose infants were initially exposed in early fetal life (n = 92, dose: median (IQR) 2.0 g (1.0–3.7)) or initially in mid–late fetal life (n = 67, dose: 2.0 g (1.0–3.5)), while a sub-group of the former group was exclusively exposed in early fetal life (n = 22, dose: 1.0 g (1.0–2.6)). The remaining 143 girls were unexposed controls. The indications of use were headache or migraine (70%), pain (predominantly from the musculoskeletal system (18%), general sickness or cold (6%), fever (5%), and other reasons (1%).

Within the exposed groups, 21 women were treated with acetylsalicylic acid for prevention of obstetrical complications from early, and throughout, pregnancy. Other non-steroidal, anti-inflammatory drugs were used by 12 women. Postpartum, 33 infants were treated with paracetamol.

Overall, the mean (±SD) maternal age at birth was 33 (4) years, and the majority of pregnant women were nulliparous (76%). There were no differences in birth weight or gestational age at birth between infants exposed during defined fetal periods and unexposed controls (Table 1). Summaries of predefined outcomes stratified by exposure groups are presented in Table 2.

**Table 1.** Maternal and infant characteristics stratified by exposure to paracetamol initially in early (< GA 17 weeks) or mid–late (GA ≥ 17 weeks) fetal life compared to unexposed controls.

	Total (n = 302)		Unexposed (n = 143)		Initially exposed in early fetal life (n = 92)		Initially exposed in mid–late fetal life (n = 67)	
	N	Mean (±SD)/median (IQR)/n (%)	N	Mean (±SD)/median (IQR)/n (%)	N	Mean (±SD)/median (IQR)/n (%)	N	Mean (±SD)/median (IQR)/n (%)
**MOTHER**								
Maternal age at delivery (years)	302	33 (4)	143	33 (4)	92	34 (4)	67	33 (4)
Maternal pre-pregnancy BMI (kg/m^2^)	294	22.3 (2.7)	138	22.2 (2.3)	89	**22.8 (3.1)** *	67	22.2 (2.3)
Nulliparous (yes)	301	228 (76)	142	110 (78)	92	65 (71)	67	54 (81)
Use of nicotine products in first trimester (yes)	302	9 (3)	143	2 (1)	92	**7 (8)** **	67	0 (0)
Alcohol consumption in first trimester (yes)	301	102 (34)	143	42 (29)	91	35 (39)	67	25 (37)
Polycystic ovarian syndrome (yes)	298	22 (7)	140	8 (6)	91	9 (10)	67	5 (8)
Mode of conception								
IUI	301	18 (6)	143	8 (6)	91	4 (4)	67	6 (9)
IVF	301	16 (5)	143	4 (3)	91	**8 (9)** *	67	4 (6)
ICSI	301	15 (5)	143	6 (4)	91	7 (8)	67	2 (3)
Dose of paracetamol (g)	159	3.0 (1.0–8.5)	143	–	92	2.0 (1.0–3.7)	67	2.0 (1.0–3.5)
**INFANT**								
Birthweight (g)	301	3489 (440)	142	3512 (412)	92	3473 (507)	67	3461 (402)
Birthweight (SDS)	301	−0.46 (0.99)	142	−0.38 (0.96)	92	−0.50 (1.15)	67	−0.56 (0.84)
Gestational age at birth (days)	302	282 (9)	143	282 (10)	92	282 (8)	67	283 (9)

GA, gestational age; BMI, body-mass index; IUI, intrauterine insemination; IVF, *in vitro* fertilization; ICSI, intracytoplasmic sperm injection; SDS, standard deviation score. *P*-values by Student’s *t*-test or Pearson chi-square test, with Fisher’s exact test applied when expected counts ≤ 5.

* Indicate associations with a *P*-value 0.05 ≤ 0.1.

** Indicate a *P*-value ≤ 0.05 and are highlighted in bold.

**Table 2.** Summaries of predefined primary and secondary outcomes in girls exposed to paracetamol initially during early fetal life (GA < 17 weeks, n = 92), and mid–late fetal life (GA ≥ 17 weeks, n = 67) compared to unexposed girls (n = 143).

	Total (n = 302)		Unexposed (n = 143)		Initially exposed in early fetal life (n = 92)		Initially exposed in mid–late fetal life (n = 67)	
	N	Mean (±SD)/median (IQR)/n (%)	N	Mean (±SD)/median (IQR)/n (%)	N	Mean (±SD)/median (IQR)/n (%)	N	Mean (±SD)/median (IQR)/n (%)
Uterine volume (cm^3^)	230	1.28 (0.50)	107	1.34 (0.60)	73	**1.18 (0.36)** **	50	1.30 (0.42)
Endometrial thickness (mm)	255	0.24 (0.06)	118	0.24 (0.07)	80	0.23 (0.06)	57	0.24 (0.06)
Ovarian volume (cm^3^)	203	0.26 (0.17–0.45)	98	0.27 (0.20–0.42)	68	0.26 (0.17–0.45)	37	0.28 (0.13–0.45)
Sum of total follicles	203	4.39 (2.10)	98	4.59 (2.22)	68	4.52 (2.11)	37	**3.57 (1.52)** **
Breast tissue diameter (cm)	300	0.87 (0.51)	142	0.89 (0.49)	91	0.88 (0.51)	67	0.81 (0.54)
Fetal AGD (cm)	295	1.28 (0.23)	139	1.27 (0.22)	90	1.27 (0.23)	66	1.30 (0.24)
AGDaf (cm)	301	1.58 (0.33)	142	1.56 (0.33)	92	1.62 (0.32)	67	1.57 (0.34)
AGDac (cm)	301	3.67 (0.48)	142	3.69 (0.46)	92	3.69 (0.51)	67	3.58 (0.47)
AMH (SDS)	262	−0.12 (1.04)	121	−0.14 (1.01)	81	−0.05 (1.13)	60	−0.20 (0.98)
Inhibin B (SDS)	256	0.03 (0.87)	118	0.02 (0.90)	78	0.16 (0.82)	60	−0.12 (0.87)
FSH (SDS)	255	−0.06 (0.88)	117	−0.07 (0.92)	79	−0.08 (0.79)	59	−0.02 (0.93)
LH measurable (yes)	255	112 (44)	117	50 (43)	79	36 (46)	59	26 (44)
Estradiol (SDS)	269	0.03 (1.04)	124	0.10 (1.05)	84	0.02 (0.98)	61	−0.09 (1.10)

GA, gestational age; SDS, standard deviation score; AGD, anogenital distance; AGDaf, anogenital distance (ano-fourchettal); AGDac, anogenital distance (ano-clitoral); LH, luteinizing hormone; FSH, follicle-stimulating hormone; AMH, anti-Müllerian hormone. *P*-values for continuous variables were obtained from univariate linear regression (Wald tests) and categorical variables from Pearson chi-square test.

** Indicate a *P*-value ≤ 0.05 and are highlighted in bold.

### Early fetal life (GA < 17 weeks)

**Figure 1.**
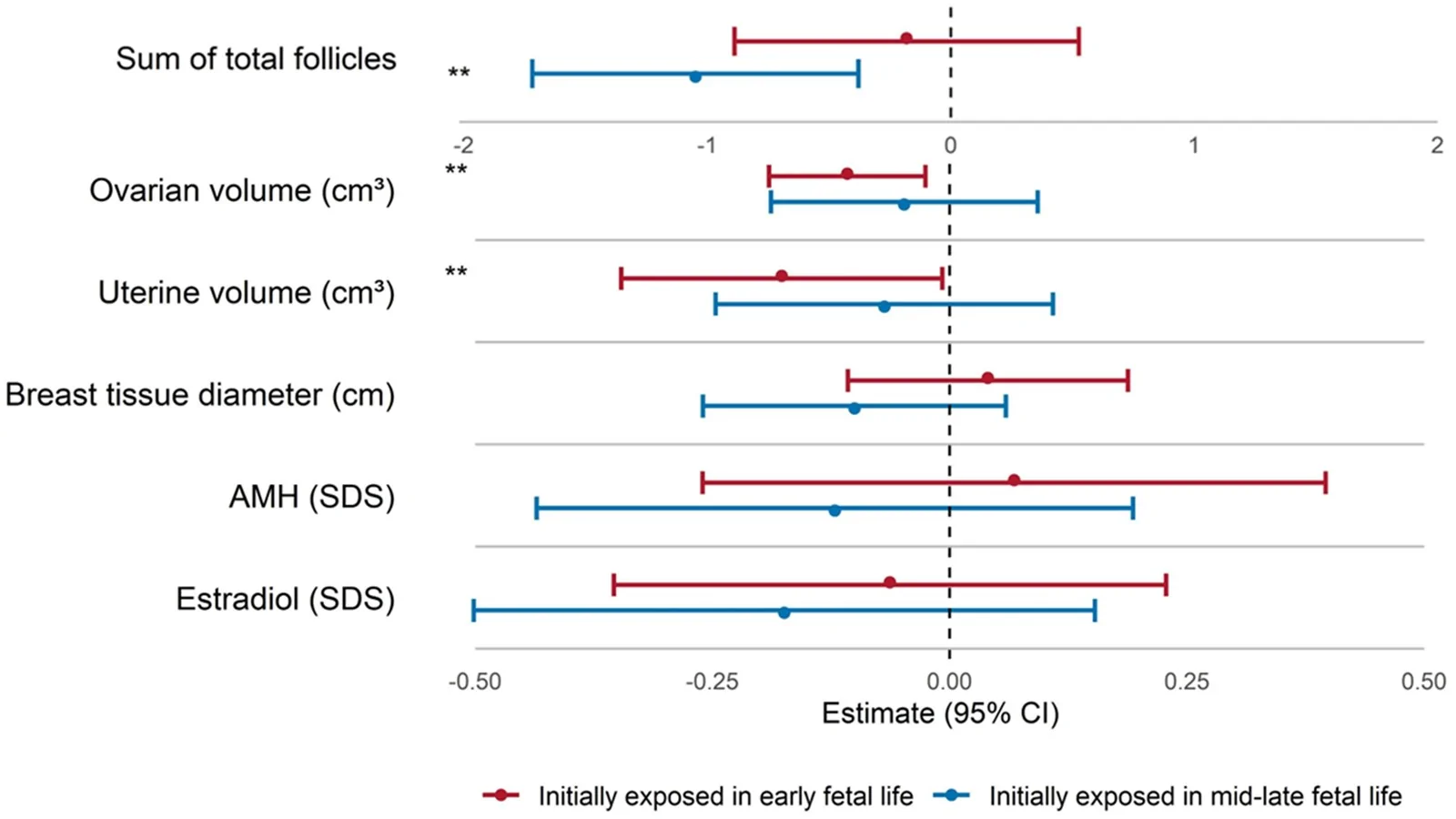
**Associations of self-reported prenatal maternal paracetamol use and reproductive outcomes in girls**. Forest plot of adjusted estimates by multiple linear regression models analyzing the association with exposure initially in early fetal life (GA < 17 weeks, n = 92) and initially during mid–late fetal life (GA ≥ 17 weeks, n = 67) compared to unexposed controls (n = 143), by maternal self-reported use or non-use of paracetamol. Estimates are adjusted for maternal polycystic ovarian syndrome status, BMI before pregnancy, alcohol and nicotine use in the first trimester, conception by IVF, maternal use of acetylic salicylic acid and other non-steroidal anti-inflammatory drugs, infant paracetamol treatment, and infant age at examination (uterine volume, breast tissue diameter). SDS, standard deviation score; AMH, anti-Müllerian hormone. Dots represent the estimated effect size, and horizontal lines indicate 95% CIs. Statistical significance was assessed using multivariable linear regression models (Wald tests), *indicates associations with a *P*-value 0.05 ≤ 0.1 and **indicates a *P*-value ≤ 0.05.

In adjusted models, girls exposed initially during early fetal life had reduced ovarian volume: β (95% CI) − 0.11 cm^3^ (−0.19 to −0.03), *P* = 0.01, corresponding to a − 41% change (−70 to −11) compared to unexposed girls. Similarly, uterine volume was reduced: −0.18 cm^3^ (−0.35 to −0.01) *P* = 0.04, corresponding to a − 13% change (−26.0 to −0.7) compared to unexposed girls (Table 3, Fig. 1).

**Table 3.** Associations between maternal self-reported use of paracetamol and predefined primary and secondary outcomes in girls exposed initially during early fetal life (GA < 17 weeks, n = 92) and mid–late fetal life (GA ≥ 17 weeks, n = 67), compared to unexposed girls (n = 143).

	Early fetal life		Mid–late fetal life	
	Unadjusted	Adjusted	Unadjusted	Adjusted
	Estimate (95% CI)	Estimate (95% CI)	Estimate (95% CI)	Estimate (95% CI)
Uterine volume (cm^3^)	−**0.16 (**−**0.30 to** −0.02)**	−**0.18 (**−**0.35 to** −0.01)**	−0.04 (−0.20 to 0.12)	−0.07 (−0.25 to 0.11)
Endometrial thickness (mm)	−0.02 (−0.20 to 0.15)	0.00 (−0.02 to 0.02)	0.01 (−0.19 to 0.20)	0.01 (−0.01 to 0.03)
Ovarian volume (cm^3^)	−0.05 (−0.14 to 0.04)	−**0.11 (**−**0.19 to** −0.03)**	−0.03 (−0.17 to 0.11)	−0.05 (−0.19 to 0.09)
Sum of total follicles	−0.06 (−0.72 to 0.60)	−0.18 (−0.88 to 0.52)	−**1.02 (**−**1.67 to** −0.37)**	−**1.05 (**−**1.71 to** −0.39)**
Breast tissue diameter (cm)	−0.01 (−0.14 to 0.12)	0.04 (−0.11 to 0.19)	−0.08 (−0.23 to 0.07)	−0.10 (−0.26 to 0.06)
Fetal AGD (cm)	0.00 (−0.06 to 0.06)	−0.03 (−0.09 to 0.03)	0.02 (−0.04 to 0.09)	0.01 (−0.05 to 0.07)
AGDaf (cm)	0.06 (−0.03 to 0.14)	0.01 (−0.12 to 0.14)	0.01 (−0.09 to 0.11)	−0.10 (−0.24 to 0.04)
AGDac (cm)	0.00 (−0.13 to 0.13)	0.05 (−0.04 to 0.14)	−**0.11 (**−0.25 to 0.02)*	−0.01 (−0.11 to 0.09)
AMH (SDS)	0.09 (−0.21 to 0.39)	0.07 (−0.26 to 0.40)	−0.06 (−0.37 to 0.24)	−0.12 (−0.43 to 0.19)
Inhibin B (SDS)	0.14 (−0.11 to 0.38)	0.18 (−0.07 to 0.43)	−0.14 (−0.41 to 0.13)	−0.14 (−0.42 to 0.14)
FSH (SDS)	−0.01 (−0.25 to 0.23)	0.01 (−0.25 to 0.27)	0.05 (−0.24 to 0.33)	0.05 (−0.25 to 0.35)
Estradiol (SDS)	−0.08 (−0.36 to 0.20)	−0.06 (−0.35 to 0.23)	−0.20 (−0.53 to 0.13)	−0.17 (−0.49 to 0.15)

Estimated mean differences with 95% CIs from linear regression models, adjusted and unadjusted. Model adjusted for maternal polycystic ovarian syndrome status, BMI before pregnancy, alcohol and nicotine use in the first trimester, conception by *in vitro* fertilization, maternal use of acetylic salicylic acid and other non-steroidal anti-inflammatory drugs, infant paracetamol treatment, and infant age at examination (uterine volume, AGDaf, AGDac, breast tissue diameter). Fetal AGD is moreover adjusted for GA at the time of ultrasound scan. SDS, standard deviation score; AGD, anogenital distance; AGDaf, anogenital distance (ano-fourchettal); AGDac, anogenital distance (ano-clitoral); FSH, follicle-stimulating hormone; AMH, anti-Müllerian hormone. Statistical significance was assessed using multivariable linear regression models (Wald tests).

* Indicate associations with a *P*-value 0.05 ≤ 0.1.

** Indicate a *P*-value ≤ 0.05 and are highlighted in bold.

Exposure exclusively in early fetal life was associated with reduced ovarian volume: −0.10 cm^3^ (−0.19 to −0.01), *P* = 0.05, as well as lower circulating levels of AMH: −0.45 SDS (−0.87 to −0.03), *P* = 0.04 (Supplementary Table S2).

### Mid–late fetal life (GA ≥ 17 weeks)

In adjusted models, girls exposed exclusively during mid–late fetal life had a reduced number of ovarian follicles: −1.05 (−1.71 follicles to −0.39), *P* < 0.01 corresponding to a − 23% (−37 to −9) change compared to unexposed girls (Table 3, Fig. 1).

Multiple regression analyses that simultaneously included both fetal exposure periods (early and mid–late) supported the hypothesis that the association between early fetal exposure to paracetamol and uterine volume was driven primarily by early fetal exposure, with no additional effect by exposure during mid–late fetal life. Similarly, reduced follicle count was associated only with mid–late fetal exposure. In these analyses, we did not see a clear association between exposure periods and ovarian volume or circulating levels of reproductive hormones (Supplementary Table S3).

### Dose–response trends

**Figure 2.**
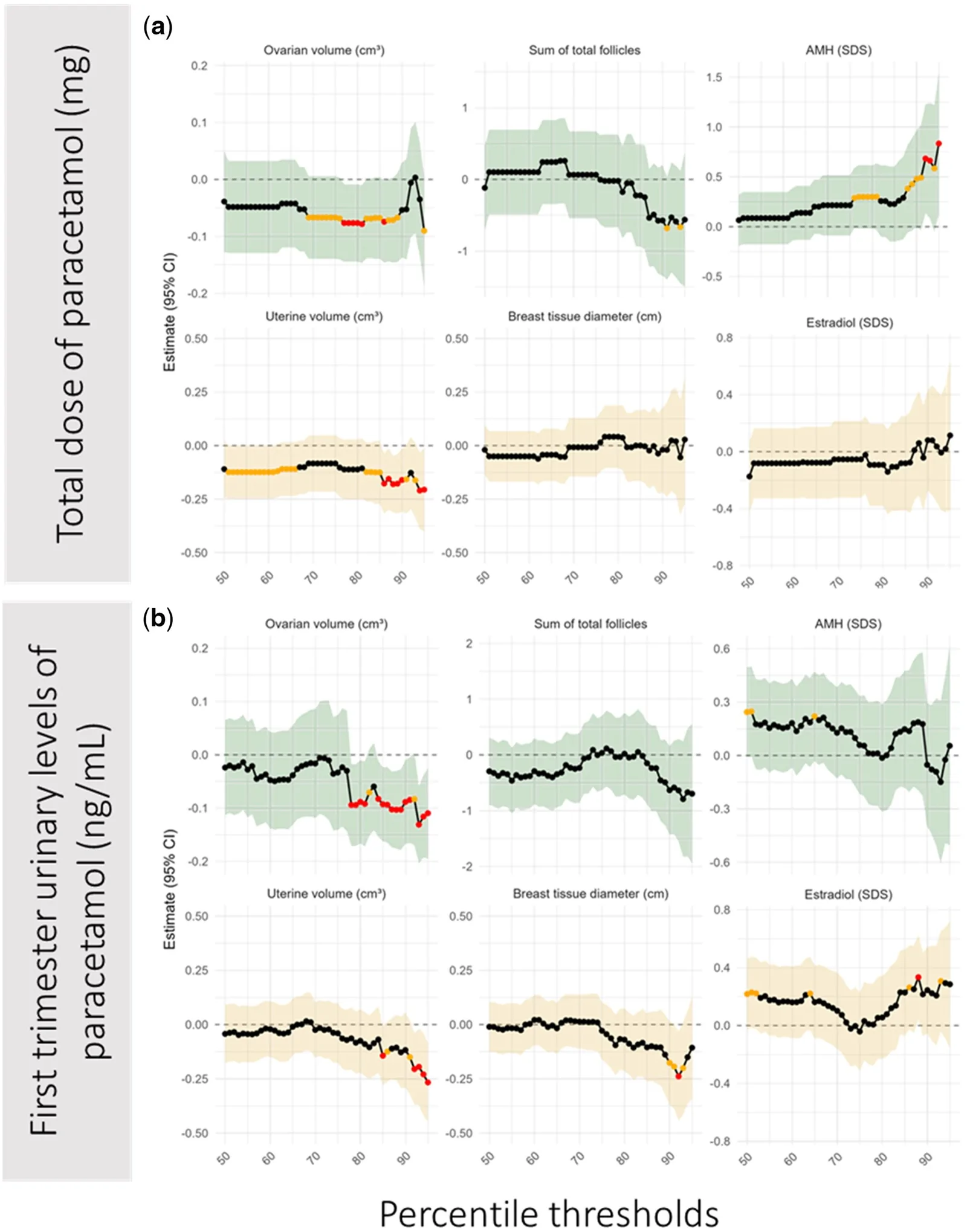
**Dose–response relationships**. Dose–response relationships for (**a**) self-reported maternal paracetamol use and (**b**) urinary paracetamol concentrations in relation to predefined primary outcomes. Figure shows effect estimates (standardized β coefficients) across the percentile thresholds comparing participants above versus below the given paracetamol exposure threshold using linear regression models. Black lines indicate regression estimates, shaded areas represent 95% CIs. Statistical significance was assessed using multivariable linear regression models (Wald tests). Points with *P* ≤ 0.05 are highlighted in red, and those with 0.05 < *P* ≤ 0.1 are highlighted in orange. Models were adjusted for maternal polycystic ovary syndrome (PCOS) status, pre-pregnancy BMI, alcohol, and nicotine use during the first trimester, conception by IVF, maternal use of acetylsalicylic acid or other non-steroidal anti-inflammatory drugs, infant paracetamol treatment, and infant age at examination (for uterine volume and breast tissue diameter outcome). SDS, standard deviation score; AMH, anti-Müllerian hormone.

The total maternal paracetamol dose during pregnancy, based on self-reported data, was median (IQR) 3.0 g (1.0–8.5). Paracetamol was measurable in all urine samples from the mothers of included girls with a median (IQR) concentration of 64.5 ng/ml (34.6–134.9) (GW mean (±SD) 14.1 (1.8)). Both total dose of self-reported paracetamol as well as maternal urinary concentrations of paracetamol were inversely associated with ovarian and uterine volume. Moreover, maternal urinary paracetamol was inversely associated with breast tissue diameter (Fig. 2). Percentile distribution of accumulated maternal self-reported doses of paracetamol use during pregnancy as well as first trimester urinary paracetamol concentrations are presented in Supplementary Table S4.

Overall, sensitivity analyses confirmed the associations between exposure to paracetamol in early or mid–late fetal life, and key outcomes were not affected by excluding infants based on covariate characteristics (data not shown) or the indication of maternal fever (Supplementary Table S5).

### Independent confirmatory cohort

In total, use of paracetamol and/or non-steroidal anti-inflammatory drugs during pregnancy was reported by 179 women (18%). Mother and infant characteristics are listed in Supplementary Table S6 and descriptive summaries of outcomes are presented in Supplementary Table S7.

At puberty, girls exposed to paracetamol and/or non-steroidal anti-inflammatory drugs during pregnancy had reduced uterine volume: −4.11 cm^3^ (−7.29 to −0.92), *P* = 0.01 as well as reduced levels of circulating inhibin B: −14.12 pg/ml (−25.20 to −3.03), *P* = 0.02, and FSH just reaching statistical significance: −0.61 IU/l (−1.21 to 0.00), *P* = 0.05. At adolescence, girls exposed to paracetamol and/or non-steroidal anti-inflammatory drugs during pregnancy had reduced ovarian volume: −2.76 cm^3^ (−4.82 to −0.70), *P* = 0.01 (Supplementary Table S8).

## Discussion

To our knowledge, this is the first prospective human cohort study designed to evaluate associations between fetal exposure to paracetamol and postnatal ovarian function. We demonstrate that even relatively low levels of fetal paracetamol exposure are associated with substantial alterations in ovarian morphology and activity. Specifically, in the COPANA cohort, girls exposed in early fetal life exhibited 40% reduction in reduced ovarian volume, whereas girls exposed in mid–late fetal life exhibited 23% reduction in follicle count. In addition, the uterine volume and breast tissue diameter were reduced after paracetamol exposure, indicating compromised estradiol production. Associations were broadly consistent and suggestive of a dose-dependent relationship across complementary exposure assessment strategies. The main results were replicated in an independent cohort followed into adolescence (although this replication was not uniform across all outcomes) suggesting that the observed alterations may persist beyond early life.

These human data align closely with a substantial body of experimental evidence. Multiple independent animal studies have consistently demonstrated reduced ovarian follicle numbers following prenatal paracetamol exposure (Dean *et al.*, 2016; Holm *et al.*, 2016; Johansson *et al.*, 2016; Rossitto *et al.*, 2019; Aleixo *et al.*, 2020; Wu *et al.*, 2024), providing biological plausibility and supporting a potential causal interpretation of the associations observed in the present study.

The observations align with our current understanding of fetal ovarian biology, and several biological mechanisms have been proposed that could explain the associations. Paracetamol is suggested to inhibit the intense mitosis of germ cells, thereby reducing the number of oogonia entering the first meiotic division (Dean *et al.*, 2016; Holm *et al.*, 2016; Hurtado-Gonzalez *et al.*, 2018; Rossitto *et al.*, 2019). This effect may occur through direct inhibition of cell proliferation, potentially via targeting ribonucleotide reductase and subsequent blockade of DNA synthesis (Nielsen *et al.*, 2025). In addition, paracetamol inhibits COX enzyme activity, leading to reduced prostaglandin synthesis and decreased expression of key pluripotency and anti-apoptotic markers in germ cells (Dean *et al.*, 2016; Hurtado-Gonzalez *et al.*, 2018; Bayne *et al.*, 2022). Moreover, reduced prostaglandin levels delay the critical transition from germ cell mitosis to meiosis resulting in intensified germ cell apoptosis, further diminishing the final pool of primordial follicles (Martins da Silva *et al.*, 2004; Dean *et al.*, 2016; Holm *et al.*, 2016; Rossitto *et al.*, 2019).

The functional establishment of the female HPG axis during minipuberty provides a unique window to assess ovarian activity (Fischer *et al.*, 2024a; Ljubicic *et al.*, 2022). AMH is produced exclusively by granulosa cells surrounding small antral follicles, and in adult women, AMH levels and the antral follicle count serve as non-invasive proxies for the size of the ovarian reserve of primordial follicles (Gougeon, 1996; Andersen *et al.*, 2010; Hansen *et al.*, 2011; Jeppesen *et al.*, 2013; Mamsen *et al.*, 2021). In adult women, age-specific AMH is therefore related to age at menopause (Depmann *et al.*, 2018). During childhood, AMH (SDS) is relatively stable from infancy to adolescence (Hagen *et al.*, 2023). In the present study, AMH levels were reduced in infant girls exposed exclusively in early fetal, suggesting that fetal paracetamol exposure may diminish the ovarian reserve. Whether these differences in early postnatal life carry long‑term clinical significance, including potential implications for the reproductive lifespan, remains to be further elucidated in future studies with extended follow‑up. In addition, ovarian volume and follicle number during female minipuberty show substantial inter-individual variability, and clinically meaningful thresholds in infancy remain poorly defined.

Further, exposure was associated with reduced sizes of estrogen-responsive tissues. At minipuberty, uterine volume and diameter of breast tissue correlate with circulating estradiol levels (Fischer *et al.*, 2024a; Kuiri-Hänninen *et al.*, 2013), and although a single estradiol concentration at a single evaluation in the present study was not associated with exposure, we speculate, that cumulative lower estradiol secretion from fewer ovarian follicles contributes to reduced growth of uterus and breast tissue.

The present study is the first human cohort specifically designed to assess associations between prenatal exposure to paracetamol and ovarian development, and exposure assessment is a critical strength of this study. We combined frequent maternal self-reported data with urinary paracetamol measurements, providing both subjective and objective exposure indices. The strong dose-response trends, particularly for ovarian and uterine volume, reinforce the robustness of the findings. Paracetamol was detected in all urine samples, reflecting widespread environmental exposure likely originating from aniline metabolism (Nielsen *et al.*, 2015; Pot *et al.*, 2022). We note that our biomarker reflects a single point-in-time measurement and is therefore primarily an indicator of recent paracetamol exposure, irrespective of whether the source is direct medication use or low-level background exposure from environmental sources. As our study was not designed to distinguish between pharmaceutical and environmental sources, we consider the measured concentrations as reflecting total exposure at a single timepoint in early fetal life. As no standardized cutoff exists for recent intake, percentile-based thresholds were applied, which yielded consistent results with reported use and suggest biological relevance even at low exposure levels.

Unique to this study is the thorough assessment of ovarian activity in infancy including detailed ovarian and uterine morphology by TAUSs, which is the most appropriate method for evaluating internal genitalia morphology in prepubertal girls.

A key strength of this study is replication of the results in an independent confirmatory cohort. Although the independent confirmatory cohort was not primarily designed to assess maternal paracetamol use, and exposure therefore is most likely highly under-reported, similar associations with ovarian and uterine morphology were observed during puberty and adolescence. In COPANA, paracetamol use was recorded prospectively every 14 days from early pregnancy, whereas exposure in the confirmatory cohort was assessed only once mid-pregnancy introducing recall bias. Previous studies further suggest that women may not consistently report paracetamol as medicine use unless specifically asked, as in the COPANA cohort (Kristensen *et al.*, 2011). Overall, these observations support the associations seen in the COPANA cohort and suggest the possibility that the observed differences may persist beyond infancy, but the clinical relevance warrants further investigation.

Hormonal assessments during adolescence did not show clear associations with exposure, likely due to menstrual cycle variability. Limited exposure data from mid-to-late pregnancy in the confirmatory cohort may have further restricted the detection of associations with follicle numbers.

Due to the observational study design, residual confounding by indication cannot be completely excluded. Maternal pain, infection, or fever may themselves influence fetal gonadal development, and detailed information on symptom severity and duration was not available. However, most reported indications for paracetamol use were headache or migraine rather than systemic illness, and sensitivity analyses excluding maternal fever and other potential confounders did not change the findings.

The number of analyses performed represents an important limitation and the possibility of type I error cannot be excluded. However, the analyses were predefined and hypothesis-driven, based on prior animal and *ex vivo* evidence suggesting effects of paracetamol on female reproductive development. Furthermore, related and biologically interconnected outcomes were affected in the same direction. We therefore did not adjust for multiple testing, which could increase the risk of Type II errors. Nevertheless, some findings were not consistently replicated across cohorts and should therefore be interpreted cautiously until confirmed in independent studies. The study population was predominantly Caucasian and term-born, which may limit generalizability. The analytic design of the current study limited our ability to evaluate whether frequency or patterns of paracetamol use influenced the observed associations; however, even with a dichotomous yes/no exposure definition across two broad exposure windows, we observed consistent and biologically plausible associations.

Ovarian volume and follicle counts were available in approximately two-thirds of infants, reflecting the challenges associated with ultrasound assessment of small infant ovaries, comparable to that reported in the few existing studies of ovarian morphology during female minipuberty (Haber and Mayer, 1994; Chin *et al.*, 2021). Associations were observed despite the limited statistical power but warrant confirmation in larger studies.

Future studies should aim to include larger prospective pregnancy cohorts with repeated biomarker-based assessment of paracetamol exposure throughout gestation and long-term follow-up into adolescence and adulthood. Predefined clinically meaningful reproductive endpoints, including fertility-related outcomes and reproductive lifespan characteristics, may help clarify the clinical implications of the observed associations.

Paracetamol is available over the counter and is widely used during pregnancy (Ersbøll *et al.*, 2015; Lind *et al.*, 2017; Taagaard *et al.*, 2023). Although the present findings show that fetal exposure was associated with ovarian morphology and activity in infancy, the extent to which these early‑life observations translate into long‑term effects on reproductive function remains to be further evaluated.

While certain clinical situations, such as severe maternal fever, require paracetamol use (Dreier *et al.*, 2014; Antoun *et al.*, 2021), current guidelines recommend minimal dosage and duration of therapy (European Medicines Agency, 2025). The exposure levels in this study fall within these recommendations, raising concern that current guidance may not be sufficient. Non-pharmacological alternatives and enhanced education for pregnant women may help reduce unnecessary exposure.

## Conclusion

In this prospective cohort study of infant girls, prenatal exposure to paracetamol was associated with differences in several markers of ovarian development, including ovarian volume, follicle number, and circulating AMH, as well as uterine volume and breast tissue diameter. Associations with reduced ovarian and uterine size were also observed in a confirmatory cohort followed to adolescence. Given the observational design, causal inference cannot be established, and clinical implications remain uncertain. The findings are consistent with experimental animal studies, however, replication in larger studies with long-term follow-up is required.

## Supplementary Material

hoag073_Supplementary_Data

## Data Availability

All data will be made available from the corresponding author at email: margit.bistrup.fischer.01@regionh.dk on reasonable request and according to GDPR rules.
