# Supplementary material for: Fetal exposure to paracetamol is associated with altered markers of ovarian development and reduced uterine volume in girls: the COPANA study
Source: Hum Reprod Open. 2026 Sep 8;2026(3):hoag073. doi: 10.1093/hropen/hoag073 (PMC13553118; doi:10.1093/hropen/hoag073)
Supplement: Supplementary file 1 [file hoag073_supplementary_data.docx]

**Supplementary material**

**Fetal exposure to paracetamol is associated with altered markers of ovarian development and reduced uterine volume in girls: the COPANA study**

**Supplementary Table S1. Hormone analyses in infant blood samples (n=269). Listed by priority of analyses.**

|  | **Method/Assay** | **Manufacturer** | **N*** | **Samples above LOD (%)** | **LOD** |
| --- | --- | --- | --- | --- | --- |
| Anti-Müllerian hormone (AMH) | Chemiluminescence immunoassay | Access2, Beckman Coulter, Brea, CA, USA | 264 | 100 | 0.14 pmol/L |
| Follicle-stimulating hormone (FSH) | Chemiluminescence  immunoassay | Atellica, Siemens NY, Healthineers, Tarrytown, USA | 264 | 100 | 0.3 IU/L |
| Luteinizing hormone (LH) | Chemiluminescence  immunoassay | Atellica, Siemens Healthi neers, Tarrytown, NY, USA | 264 | 47 | 0.07 IU/L |
| Inhibin B | Enzyme-linked immunosorbent assay | Immunotech, Beckman Coulter, Brea, CA, USA | 265 | 97 | 3 ng/L |
| Estradiol | LC/MS-MS | *In-house* | 265 | 89 | 4.0 pmol/L |
| Progesterone | LC/MS-MS | *In-house* | 269 | 100 | 0.036 nmol/L |
| Androstenedione | LC/MS-MS | *In-house* | 269 | 100 | 0.031 nmol/L |
| Testosterone | LC/MS-MS | *In-house* | 269 | 100 | 0.031 nmol/L |
|  |  |  |  |  |  |

LOD: limit of detection, LC/MS-MS: liquid chromatography-tandem mass spectrometry. *Because the analyses require varying amounts of sample volume, the number of completed tests does not always align with the prioritization.

| **Supplementary Table S2. Associations between maternal self-reported use of paracetamol and predefined primary and secondary outcomes in girls exposed exclusively during early fetal life (GA < 17 weeks, n = 22) compared to unexposed girls (n = 143).** | | |
| --- | --- | --- |
|  | **Unadjusted** | **Adjusted** |
|  | **Estimate (95% CI)** | **Estimate (95% CI)** |
| Uterine volume (cm³) | **−0.19 (−0.39; 0.01)*** | **−0.2 (−0.43; 0.03)*** |
| Endometrial thickness (mm) | −0.10 (−0.35; 0.16) | 0.00 (−0.03; 0.03) |
| Ovarian volume (cm³) | **−0.12 (−0.22; −0.02)**** | **−0.10 (−0.19; −0.01)**** |
| Sum of total follicles | −0.09 (−1.32; 1.13) | 0.15 (−1.1; 1.40) |
| Breast tissue diameter (cm) | 0.02 (−0.21; 0.24) | −0.04 (−0.26; 0.18) |
| Fetal AGD (cm) | 0.02 (−0.09; 0.14) | 0.00 (−0.11; 0.11) |
| AGDac (cm) | 0.10 (−0.10; 0.31) | −0.01 (−0.17; 0.15) |
| AGDaf (cm) | 0.02 (−0.15; 0.20) | 0.06 (−0.14; 0.26) |
| AMH (SDS) | **−0.41 (−0.83; 0.00)**** | **−0.45 (−0.87; −0.03)**** |
| Inhibin B (SDS) | 0.11 (−0.35; 0.58) | 0.13 (−0.33; 0.59) |
| FSH (SDS) | 0.33 (−0.10; 0.75) | **0.39 (−0.07; 0.85)*** |
| Estradiol (SDS) | −0.27 (−0.76; 0.22) | −0.24 (−0.72; 0.24) |
| Estimated mean differences with 95% confidence intervals (CI) from linear regression models, adjusted and unadjusted. Model adjusted for maternal polycystic ovarian syndrome status, BMI before pregnancy, alcohol and nicotine use in the first trimester, conception by in vitro fertilization, maternal use of acetylic salicylic acid and other non-steroidal inflammatory drugs, infant paracetamol treatment and infant age at examination (uterine volume, AGDaf, AGDac, breast tissue diameter). Fetal AGD is moreover adjusted for GA at time of ultrasound scan. SDS: standard deviation score, AGD: anogenital distance, AGDaf: anogenital distance (ano-fourchettal), AGDac: anogenital distance (ano-clitoral), FSH: follicle stimulating hormone, AMH: anti-Müllerian hormone. Statistical significance was assessed using multivariable linear regression models (Wald tests), *indicate associations with a p-value 0.05 ≤ 0.1 and **indicate a p-value ≤ 0.05 and are highlighted in bold. | | |

| **Supplementary Table S3. Multiple regression analyses estimating the independent association of each exposure period between fetal exposure to paracetamol (yes/no) in girls exposed during early fetal life (GA < 17 weeks, n = 92) and/or during mid–late fetal life (GA ≥ 17 weeks, n = 137).** | |
| --- | --- |
|  | **Estimate (95% CI)** |
| Fetal AGD (cm) |  |
| Paracetamol in early fetal life (GA < 17 weeks) | −0.015 (−0.080; 0.050) |
| Paracetamol in mid–late fetal life (GA ≥ 17 weeks) | 0.008 (−0.052; 0.068) |
| Uterine volume (cm³) |  |
| Paracetamol in early fetal life (GA < 17 weeks) | −**0.141 (**−**0.26;** −**0.022)**** |
| Paracetamol in mid–late fetal life (GA ≥ 17 weeks) | −0.018 (−0.145; 0.109) |
| Endometrial thickness (mm) |  |
| Paracetamol in early fetal life (GA < 17 weeks) | −0.039 (−0.200; 0.122) |
| Paracetamol in mid–late fetal life (GA ≥ 17 weeks) | 0.032 (−0.127; 0.191) |
| Ovarian volume (cm³) |  |
| Paracetamol in early fetal life (GA < 17 weeks) | −0.042 (−0.144; 0.060) |
| Paracetamol in mid–late fetal life (GA ≥ 17 weeks) | 0.003 (−0.105; 0.111) |
| Sum of total follicles |  |
| Paracetamol in early fetal life (GA < 17 weeks) | **0.621 (**−**0.027; 1.269)*** |
| Paracetamol in mid–late fetal life (GA ≥ 17 weeks) | −**0.735 (**−**1.332;** −**0.138)**** |
| AGDaf (cm) |  |
| Paracetamol in early fetal life (GA < 17 weeks) | 0.046 (−0.047; 0.139) |
| Paracetamol in mid–late fetal life (GA ≥ 17 weeks) | 0.020 (−0.066; 0.106) |
| AGDac (cm) |  |
| Paracetamol in early fetal life (GA < 17 weeks) | 0.087 (−0.042; 0.216) |
| Paracetamol in mid–late fetal life (GA ≥ 17 weeks) | −**0.119 (**−**0.234;** −**0.004)**** |
| Breast tissue diameter (cm) |  |
| Paracetamol in early fetal life (GA < 17 weeks) | 0.049 (−0.090; 0.188) |
| Paracetamol in mid–late fetal life (GA ≥ 17 weeks) | −0.068 (−0.196; 0.060) |
| AMH (SDS) |  |
| Paracetamol in early fetal life (GA < 17 weeks) | 0.059 (−0.235; 0.353) |
| Paracetamol in mid–late fetal life (GA ≥ 17 weeks) | 0.120 (−0.143; 0.383) |
| Inhibin B (SDS**)** |  |
| Paracetamol in early fetal life (GA < 17 weeks) | **0.225 (**−**0.024; 0.474)*** |
| Paracetamol in mid–late fetal life (GA ≥ 17 weeks) | −0.097 (−0.333; 0.139) |
| FSH (SDS) |  |
| Paracetamol in early fetal life (GA < 17 weeks) | 0.004 (−0.246; 0.254) |
| Paracetamol in mid–late fetal life (GA ≥ 17 weeks) | −0.075 (−0.319; 0.169) |
| Estradiol (SDS) |  |
| Paracetamol in early fetal life (GA < 17 weeks) | 0.020 (–0.273; 0.313) |
| Paracetamol in mid–late fetal life (GA ≥ 17 weeks) | −0.084 (−0.364; 0.196) |
| Estimated mean differences with 95% confidence intervals (CI) from linear regression models. Model adjusted for maternal polycystic ovarian syndrome status, BMI before pregnancy, alcohol and nicotine use in the first trimester, conception by in vitro fertilization, maternal use of acetylic salicylic acid and other non-steroidal anti-inflammatory drugs, infant paracetamol treatment and infant age at examination (uterine volume, AGDaf, AGDac, breast tissue diameter). SDS: standard deviation score, FSH: follicle stimulating hormone, AGDaf: anogenital distance (ano-fourchettal), AGDac: anogenital distance (ano-clitoral), AMH: anti-Müllerian hormone. Statistical significance was assessed using multivariable linear regression models (Wald tests), *indicate associations with a p-value 0.05 ≤ 0.1 and **indicate a p-value ≤ 0.05 and are highlighted in bold. | |

| **Supplementary Table S4.** **Percentile distribution of accumulated maternal self-reported doses of paracetamol use during pregnancy (n = 302) as well as first trimester urinary paracetamol (U-paracetamol) concentrations (n = 299).** | | | | |
| --- | --- | --- | --- | --- |
|  | **Dose of paracetamol** | | **U-paracetamol** | |
| Percentile | mg | n | ng/mL | n |
| 50 | 750.0 | 151/151 | 64.4 | 149/150 |
| 75 | 3375.0 | 226/76 | 134.9 | 224/75 |
| 95 | 20000.0 | 285/17 | 3008.6 | 284/15 |
|  |  |  |  |  |

| **Supplementary Table S5. Associations between maternal self-reported paracetamol use and predefined primary and secondary outcomes in girls exposed during early fetal life (GA < 17 weeks, n = 92) or during mid–late fetal life (GA ≥ 17 weeks, n = 67), compared to unexposed girls (n = 143). All analyses exclude infants whose mothers reported fever as an indication for paracetamol use.** | | | |
| --- | --- | --- | --- |
|  | **Early fetal life** | **Mid–Late fetal life** | |
|  | **Estimate (95% CI)** | | **Estimate (95% CI)** |
| Uterine volume (cm³) | −0.12 (−0.29; 0.05) | | −0.13 (−0.40; 0.14) |
| Endometrial thickness (mm) | −0.04 (−0.24; 0.15) | | −0.07 (−0.38; 0.24) |
| Ovarian volume (cm³) | −0.05 (−0.15; 0.06) | | −0.07 (−0.27; 0.13) |
| Sum of total follicles | −0.05 (−0.15; 0.06) | | −**1.66 (**−**2.96;** −**0.35)**** |
| Breast tissue diameter (cm) | 0.06 (−0.08; 0.20) | | −0.09 (−0.29; 0.11) |
| Fetal AGD (cm) | 0.01 (−0.05; 0.07) | | 0.00 (−0.07; 0.07) |
| AGDaf (cm) | 0.05 (−0.04; 0.14) | | −0.01 (−0.15; 0.12) |
| AGDac (cm) | 0.03 (−0.11; 0.17) | | −0.12 (−0.31; 0.07) |
| AMH (SDS) | 0.15 (−0.17; 0.47) | | −0.09 (−0.54; 0.36) |
| Inhibin B (SDS) | 0.07 (−0.19; 0.34) | | −0.27 (−0.66; 0.12) |
| FSH (SDS) | −0.04 (−0.31; 0.23) | | 0.00 (−0.42; 0.42) |
| Estradiol (SDS) | −0.08 (−0.39; 0.22) | | −0.32 (−0.78; 0.13) |
| Estimated mean differences with 95% confidence intervals (CI) from linear regression models. SDS: standard deviation score, AGD: anogenital distance, AGDaf: anogenital distance (ano-fourchettal), AGDac: anogenital distance (ano-clitoral), FSH: follicle stimulating hormone, AMH: anti-Müllerian hormone. Statistical significance was assessed using multivariable linear regression models (Wald tests), *indicate associations with a p-value 0.05 ≤ 0.1 and **indicate a p-value ≤ 0.05 and are highlighted in bold. | | | |

| **Supplementary Table S6. Maternal and infant characteristics stratified by exposure to paracetamol and/or NSAIDs in fetal life (n = 179) compared to unexposed controls (n=1,012) from the Copenhagen mother-child cohort.** | | | | | | |
| --- | --- | --- | --- | --- | --- | --- |
|  |  |  |  |  |  |  |
|  | **Total** | | **Unexposed** | | **Exposed** | |
|  | **N** | **Mean (±SD) / n (%)** | **N** | **Mean (±SD) / n (%)** | **N** | **Mean (±SD) / n (%)** |
| Gestational age at birth (weeks) | 1142 | 39.63 (1.95) | 964 | 39.63 (1.95) | 178 | 39.64 (1.95) |
| Maternal thyroid disease or diabetes | 1191 | 24 (2) | 1012 | 19 (2) | 179 | 5 (3) |
| Maternal smoking | 1077 | 260 (24) | 906 | 230 (25) | 171 | **30 (18)**** |
| Birth weight (kg) | 879 | 3.43 (0.55) | 734 | 3.41 (0.53) | 145 | **3.52 (0.61)**** |
| Birth length (cm) | 876 | 50.57 (2.24) | 731 | 50.51 (2.22) | 145 | **50.90 (2.32)*** |
| Infancy | 1083 |  | 904 |  | 179 |  |
| Age (days) | 1076 | 101.11 (24.01) | 897 | 101.41 (24.14) | 179 | 99.61 (23.35) |
| BMI (kg/m²) | 1069 | 16.24 (1.46) | 891 | 16.22 (1.46) | 178 | 16.33 (1.45) |
| Puberty | 563 |  | 456 |  | 107 |  |
| Age (years) | 563 | 10.72 (1.56) | 456 | 10.72 (1.65) | 107 | 10.71 (1.14) |
| BMI (kg/m²) | 533 | 17.37 (2.59) | 432 | 17.29 (2.51) | 101 | 17.71 (2.91) |
| Tanner breast stage 1 | 118 | 15 (13) | 96 | 13 (14) | 22 | 2 (9) |
| Tanner breast stage 2 | 118 | 31 (27) | 96 | 25 (26) | 22 | 6 (27) |
| Tanner breast stage 3 | 118 | 34 (29) | 96 | 24 (25) | 22 | **10 (46)*** |
| Tanner breast stage 4 | 118 | 36 (31) | 96 | 32 (33) | 22 | 4 (18) |
| Tanner breast stage 5 | 118 | 2 (2) | 96 | 2 (2) | 22 | 0 (0) |
| Adolescence | 317 |  | 266 |  | 51 |  |
| Age (years) | 317 | 16.05 (0.89) | 266 | 16.10 (0.93) | 51 | **15.78 (0.59)**** |
| BMI (kg/m²) | 314 | 21.52 (3.04) | 263 | 21.54 (3.10) | 51 | 21.46 (2.74) |
| AMH: anti-Müllerian hormone, FSH: follicle stimulating hormone, LH: luteinizing hormone. P-values for continuous variables were obtained from a Student's t-test. Girls using hormonal contraceptives are removed from adolescent analyses. *indicate associations with a p-value of 0.05 ≤ 0.1 and **indicate a p-value ≤ 0.05 and are highlighted in bold. | | | | | | |
|  |  |  |  |  |  |  |

| **Supplementary Table S7. Summaries of outcomes in girls exposed during pregnancy (n = 179) compared to unexposed girls (n = 1,012) from the Copenhagen mother-child cohort.** | | | | | | |
| --- | --- | --- | --- | --- | --- | --- |
|  |  |  |  |  |  |  |
|  | **Total** | | **Unexposed** | | **Exposed** | |
|  | **N** | **Mean (±SD)** | **N** | **Mean (±SD)** | **N** | **Mean (±SD)** |
| Infancy | 1083 |  | 904 |  | 179 |  |
| AMH (pmol/L) | 442 | 17.18 (14.25) | 370 | 16.82 (13.23) | 72 | 19.06 (18.65) |
| Inhibin B (pg/ml) | 552 | 81.86 (51.24) | 466 | 83.15 (51.53) | 86 | 74.86 (49.33) |
| FSH (IU/L) | 587 | 4.99 (4.25) | 496 | 4.97 (4.03) | 91 | 5.12 (5.29) |
| Puberty | 563 |  | 456 |  | 107 |  |
| AMH (pmol/L) | 399 | 23.36 (14.62) | 320 | 23.64 (14.83) | 79 | 22.25 (13.77) |
| Inhibin B (pg/ml) | 398 | 42.42 (28.16) | 319 | 43.37 (28.75) | 79 | 38.59 (25.41) |
| FSH (IU/L) | 398 | 3.29 (1.94) | 319 | 3.30 (1.97) | 79 | 3.27 (1.81) |
| LH (IU/L) | 398 | 1.61 (2.64) | 319 | 1.68 (2.82) | 79 | 1.31 (1.68) |
| MRI: Uterine volume (cm^3^) | 61 | 18.16 (15.10) | 53 | 19.47 (15.40) | 8 | **9.46 (9.63)**** |
| MRI: Ovarian volume (cm^3^) | 84 | 6.91 (3.81) | 67 | 7.05 (4.02) | 17 | 6.40 (2.93) |
| MRI: Sum of total follicles | 87 | 20.99 (10.51) | 70 | 21.10 (10.81) | 17 | 20.53 (9.45) |
| Adolescence | 317 |  | 266 |  | 51 |  |
| AMH (pmol/L) | 305 | 28.05 (16.54) | 253 | 28.16 (16.77) | 52 | 27.52 (15.49) |
| Inhibin B (pg/ml) | 294 | 66.96 (37.03) | 245 | 67.49 (37.72) | 49 | 64.29 (33.63) |
| FSH (IU/L) | 294 | 4.91 (2.57) | 245 | 4.81 (2.62) | 49 | 5.38 (2.28) |
| LH (IU/L) | 294 | 4.10 (5.80) | 245 | 3.90 (5.53) | 49 | 5.12 (6.97) |
| TAUS: Uterine volume (cm^3^) | 288 | 46.87 (12.44) | 240 | 47.03 (12.57) | 48 | 46.06 (11.85) |
| TAUS: Ovarian volume (cm^3^) | 288 | 15.92 (11.32) | 239 | 16.30 (12.27) | 49 | **14.07 (4.09)**** |
| TAUS: Sum of total follicles | 287 | 19.17 (4.37) | 239 | 19.12 (4.32) | 48 | 19.46 (4.64) |
| AMH: anti-Müllerian hormone, FSH: follicle stimulating hormone, LH: luteinizing hormone, MRI: Magnetic Resonance Imaging, TAUS: transabdominal ultrasound scan. P values for continuous variables were obtained from a Student's t-test. Girls using hormonal contraceptives are removed from adolescent analyses.**indicate a p-value ≤ 0.05 and are highlighted in bold. | | | | | | |
|  |  |  |  |  |  |  |

| **Supplementary Table S8. Adjusted and unadjusted associations between maternal self-reported use of paracetamol and/or non-steroidal anti-inflammatory drugs in girls exposed during pregnancy (n = 179) compared to unexposed girls (n = 1,012) from the Copenhagen mother-child cohort.** | | |
| --- | --- | --- |
|  | **Unadjusted** | **Adjusted** |
|  | **Estimate (95% CI)** | **Estimate (95% CI)** |
| Infancy |  |  |
| AMH (pmol/L) | 2.24 (−2.25; 6.72) | 2.29 (−2.11; 6.69) |
| Inhibin B (pg/ml) | −8.29 (−19.66; 3.08) | −8.03 (−19.21; 3.15) |
| FSH (IU/L) | 0.16 (−0.98; 1.29) | 0.16 (−0.98; 1.30) |
| Puberty |  |  |
| AMH (pmol/L) | −1.38 (−4.81; 2.04) | 1.97 (−4.08; 8.03) |
| Inhibin B (pg/ml) | −4.77 (−11.17; 1.63) | −**14.12 (**−**25.20;** −**3.03)**** |
| FSH (IU/L) | −0.03 (−0.49; 0.42) | −**0.61 (**−**1.21;** −**0.00)**** |
| LH (IU/L) | −0.37 (−0.85; 0.11) | −0.37 (−0.88; 0.14) |
| MRI: Uterine volume (cm^3^) | −**10.01 (**−**17.49;** −**2.54)**** | −**4.11 (**−**7.29;** −**0.92)**** |
| MRI: Ovarian volume (cm^3^) | −0.64 (−2.30; 1.01) | −0.05 (−1.33; 1.23) |
| MRI: Sum of total follicles | −0.57 (−5.60; 4.47) | −0.36 (−5.21; 4.50) |
| Adolescence |  |  |
| AMH (pmol/L) | −0.64 (−5.29; 4.01) | −1.19 (−6.42; 4.03) |
| Inhibin B (pg/ml) | −3.20 (−13.65; 7.24) | −5.69 (−17.06; 5.67) |
| FSH (IU/L) | 0.57 (−0.14; 1.28) | 0.46 (−0.28; 1.21) |
| LH (IU/L) | 1.22 (−0.84; 3.27) | 1.12 (−1.08; 3.32) |
| TAUS: Uterine volume (cm^3^) | −0.97 (−4.65; 2.71) | −0.59 (−4.40; 3.22) |
| TAUS: Ovarian volume (cm^3^) | −**2.23 (**−**4.15;** −**0.31)**** | −**2.76 (**−**4.82;** −**0.70)**** |
| TAUS: Sum of total follicles | 0.34 (−1.07; 1.75) | 0.17 (−1.31; 1.65) |
| Estimated mean differences with 95% confidence intervals (CI) from linear regression models. Model adjusted for maternal thyroid disease or diabetes and gestational week at birth. Further, pubertal outcomes are adjusted for Tanner stage at time of examination and girls using hormonal contraceptives are removed from adolescent analyses. AMH: anti-Müllerian hormone, FSH: follicle stimulating hormone, LH: luteinizing hormone, MRI: Magnetic Resonance Imaging, TAUS: transabdominal ultrasound scan. Statistical significance was assessed using multivariable linear regression models (Wald tests), **indicate a p-value ≤ 0.05 and are highlighted in bold. | | |

**Supplementary Figure S1**. **Study timeline showing study visits, questionnaires and sample collection**

GW: gestational week

Q1: Routine web-based questionnaire

Q2: Study specific questionnaire on general- and reproductive health

Q3: Study specific questionnaire on medical use every two weeks
